# Schizophrenia as a Disorder of Biological Barriers: A Narrative Review and Potential Interventions

**DOI:** 10.3390/ijms27041811

**Published:** 2026-02-13

**Authors:** Adonis Sfera, Nyla Jafri, Jacob Anton, Dragos Turturica, Edelina Turturica, Bernardo Bozza, Ioana Ciuperca

**Affiliations:** 1 Department of Psychiatry, Loma Linda University, Patton State Hospital, Patton, CA 92369, USA; 2 San Juan Bautista School of Medicine, Caguas 00727, Puerto Rico; 3 Department of Biological Sciences, California Baptist University, Riverside, CA 92504, USA; 4 Department of Psychiatry, College Hospital, Long Beach, CA 90813, USA; 5 Department of Psychiatry, Sapienza University, 00185 Rome, Italy; 6 Department of Psychiatry, University of California Los Angeles (UCLA), Los Angeles, CA 90095, USA

**Keywords:** cellular senescence, aryl hydrocarbon receptor, schizophrenia, gray matter volume

## Abstract

Severe mental illnesses, including schizophrenia and schizophrenia-like disorders, have been associated with premature neuronal and glial senescence, microglial activation, and gray matter volume reduction. These changes may drive clinical symptoms of schizophrenia, including cognitive impairment. Aryl hydrocarbon receptor, abundantly expressed in the intestinal and blood–brain barrier, is the master regulator of both tight junctions and cellular senescence. Under pathological circumstances, this receptor may promote premature gut aging, enabling the translocation of bacteria or their components from the gastrointestinal tract into systemic circulation and from there into the central nervous system. In this review article, we discuss a potential mechanism of schizophrenia–microorganismal migration, microglial activation, and gray matter volume reduction. We also focus on potential interventions for maintaining barrier function. These approaches include natural and synthetic modulators of the aryl hydrocarbon receptor as well as biophysical strategies to preserve barrier integrity and prevent central nervous system pathology.

## 1. Introduction

Schizophrenia (SCZ) is characterized by the reduction of central nervous system (CNS) gray matter volume that is detected by numerous neuroimaging methods such as positron emission tomography (PET) and magnetic resonance imaging (MRI). The gray matter changes originate in the parietal and temporal cortices and spread gradually throughout the cerebral hemispheres [1]. Gray matter volume depletion, a hallmark of severe mental illness (SMI), including SCZ, accelerates with age and may be driven by activated microglia and the aberrant phagocytosis of healthy neurons and synapses [2,3]. Since both medicated and antipsychotic-naïve patients exhibit gray matter depletion, this phenomenon may be unrelated to psychotropic drugs and reflect the primary SCZ pathology.

In this review, we construe that increased intestinal permeability allows microbial molecules to migrate from the gut lumen into the peripheral circulation and activate macrophages and microglia, leading to the elimination of healthy neurons and subsequent reduction in gray matter volume.

Several studies have detected elevated peripheral blood microbial translocation markers, including soluble CD14 (sCD14) and lipopolysaccharide-binding protein (LBP), in many patients with SCZ, indicating increased intestinal permeability and the likely migration of microbes or bacterial components into systemic circulation [4,5]. In addition, inflammatory bowel disease (IBD) (a condition marked by high microbial migration outside the gastrointestinal (GI) tract) is often comorbid with SCZ, linking bacterial molecules, such as lipopolysaccharide (LPS), to this disorder [6,7,8,9]. Moreover, infection with human immunodeficiency virus (HIV), associated with the excessive translocation of intestinal microbes into the circulatory system, presents with SCZ comorbidity, further emphasizing the role of bacteria and their components in the pathogenesis of this disorder [10,11,12].

Recent studies have shown that the aryl hydrocarbon receptor (AhR) plays a major role in both IBD and HIV infection, linking this transcription factor to the extraintestinal migration of microbes and/or their molecules [13,14,15,16]. AhR is abundantly expressed in the gut barrier and blood–brain barrier (BBB) where it modulates permeability by directly interacting with microbial molecules and tight junctions (TJs) [17,18]. Indeed, AhR ligands, such as microbial molecules, regulate cellular senescence in many cell types, including intestinal epithelial cells (IECs), neurons, and glia [19,20,21].

In previous articles, we discussed the role of dysfunctional AhR in the etiopathogenesis of SCZ [22,23]. Here, we focus on specific molecular drivers, such as the S100A9 protein, and their role in cellular senescence, microglial activation, and gray matter volume reduction [24,25]. S100A9, also known as calgranulin B, is a recently identified SCZ biomarker that, upon release in the extracellular compartment, acts as a damage-associated molecular pattern (DAMP), activating microglia via Toll-like receptor 4 (TLR4) [26,27]. Therefore, gray matter reduction in SCZ is likely triggered by S100A9-activated microglia and the aberrant elimination of healthy neurons and synapses [25,28]. Indeed, brain cell cultures exposed to S100A9 exhibit the externalization of phosphatidylserine (PS), an established “eat me” signal, followed by phagocytic neuronal loss [29]. Moreover, premature cellular senescence generates local inflammation via senescence-associated secretory phenotype (SASP), a proinflammatory secretome (that often contains S100A9), promoting further senescence and excessive phagocytosis of healthy synapses and neurons [30,31,32].

Aside from discussing the etiopathogenetic mechanisms of gray matter loss in SCZ, we also focus on natural and synthetic modulators of AhR as well as on biophysical strategies for averting CNS pathology by preserving the gut barrier and BBB.

## 2. The Microbiome and AhR

**Figure 1:**
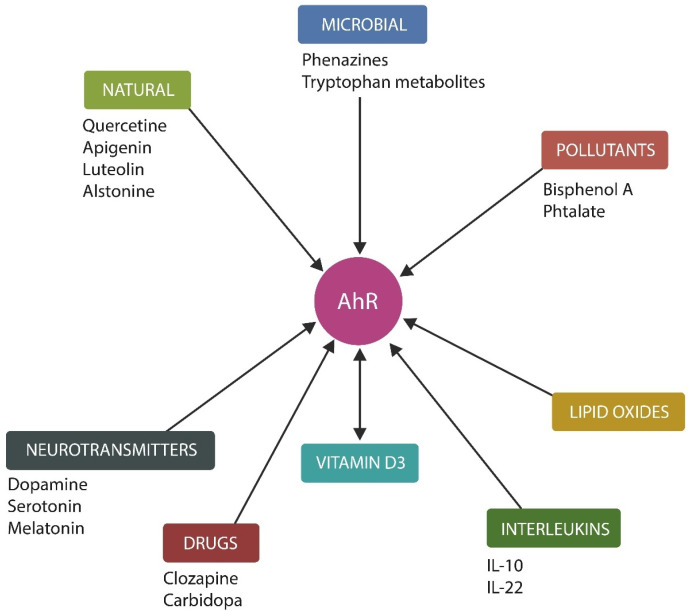
AhR ligands relevant to neuropathology. Microbial and synthetic phenazines, pollutants, lipid peroxidation derivatives, and nutrients are AhR modulators. In addition, neurotransmitters, psychotropic drugs (including clozapine and carbidopa), vitamin D, and interleukin 10 and 22 are newly identified AhR ligands with relevance to neuropsychiatry.

The human GI tract harbors approximately 100 trillion microbes belonging to more than 1000 species, comprising a complex ecosystem that contributes to health and disease by releasing metabolites implicated in numerous cellular functions [33]. Many of these molecules exert their actions via AhR, a ligand-activated transcription factor, expressed in both the gut barrier and neurovascular unit [34]. AhR belongs to the basic helix–loop–helix/Per-Arnt-Sim (bHLH/PAS) superfamily of sensors for exogenous and endogenous ligands and participates in the metabolism of xenobiotics. Over the past three decades, several non-xenobiotic AhR ligands have been identified and found to play a crucial role in the processing of psychoactive molecules, including dopamine (DA), tryptophan, and vitamin D (Figure 1). Indeed, several therapeutics and neurotransmitters relevant for SMI are AhR ligands, likely emphasizing the role of this receptor in neuropathology (Figure 1). In addition, AhR plays an essential role in driving IEC senescence, an emerging field, intersecting with both gut and brain pathology [35]. This is relevant because the gut microbiota is immunologically tolerated in the intestinal lumen, but triggers inflammation (by inflammasome activation) upon translocation into systemic circulation. SASP-induced inflammation promotes further cellular senescence and barrier disruption, facilitating the migration of microbes or their components outside the GI tract [36]. Moreover, as commensal gut bacteria generate AhR-activating metabolites implicated in neuropathology, such as tryptophan, serotonin (5HT) and melatonin, a dysfunction of this receptor may explain the comorbidity of IBD with SMI (Figure 1). Along these lines, a recent preclinical study has demonstrated that previous intestinal inflammations are recorded in the insular cortex (IC), a brain area involved in SMI, emphasizing that gut and brain pathology may form a continuum [37].

Upon migration into systemic circulation, gut microbes can promote neuronal senescence by releasing various proinflammatory molecules, including LPS [38]. For example, a preclinical study found that microbial trimethylamine-N-oxide (TMAO) could induce cellular senescence and cognitive decline in rodents, linking this pathology to molecular aging [39]. In addition, a high concentration of brain kynurenine, a tryptophan metabolite, was demonstrated to induce neuronal senescence via AhR activation [40]. In microglia, excessive kynurenine was associated with the aberrant elimination of healthy neurons and synapses, likely contributing to gray matter volume reduction [41]. Moreover, microbiome-derived indole-3-lactic acid was shown to activate microglial AhR, leading to the phagocytosis of viable neurons and glia [42]. Along these lines, lactylated histone H3K18 in microglia was demonstrated to induce neurotoxicity, marked by the elimination of healthy synapses and neurons [43,44,45,46,47]. Since SCZ was previously linked to increased brain lactate and the alteration of histone proteins, it is likely that lactylated neurotoxic microglia may contribute to the pathogenesis of SMI [48,49,50]. Furthermore, S100A9 can increase lactic acid by upregulating glycolysis, further contributing to gray matter volume reduction by pathologically activated microglia [51,52,53].

Along these lines, several studies have found that light can modulate AhR via the photo-oxidation of tryptophan metabolites and other endogenous ligands [54]. For example, blue and ultraviolet radiation (UVR) can increase AhR activity, highlighting a previously described link between UVR and SCZ [55]. Moreover, other AhR ligands, including pollutants such as polycyclic aromatic hydrocarbons (PAHs), phthalate, and bisphenol A, were associated with SCZ, further implicating AhR in the pathogenesis of this disorder [56]. Interestingly, both UVR and PAH have been shown to increase the expression of S100A9, likely connecting this SCZ marker to the reduction of gray matter volume [57,58].

### 2.1. AhR and Inflammasomes

SCZ has been associated with inflammasome activation in brain cells, including microglia. Several postmortem studies have demonstrated increased levels of inflammasome components, such as NLRP3, in the brains of patients with SCZ, implicating neuroinflammation in the pathogenesis of this disorder [59]. In addition, elevated inflammasome-linked interleukins, interleukin 1 beta (IL-1 β) and interleukin-18 (IL-18), were documented in SCZ, further connecting this condition to neuroinflammation [60].

**Figure 2:**
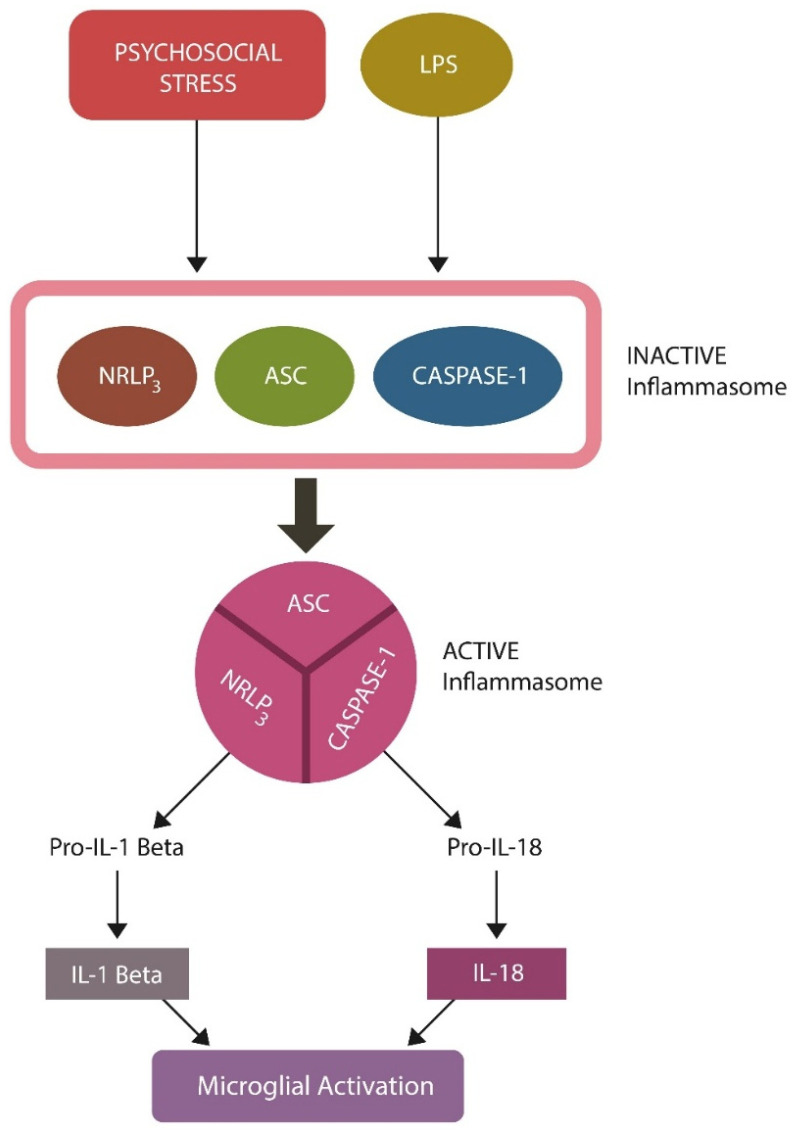
Inflammasome activation: a two-step process. The inflammasome is a protein complex comprising NRLP3, ASC, and caspase-1. Microbial antigens, including LPS, or psychosocial stress, comprise the “first signal” that primes the inactive inflammasome (unattached NLRP3, ASC, and caspase 1). A “second signal”, such as excessive ROS or calcium, is necessary to fully activate inflammasomes and trigger inflammation. Active inflammasomes generate IL-1β and IL-18, activating microglia. Aberrant microglial activation can trigger the phagocytosis of healthy neurons and synapses, leading to gray matter volume reduction.

Cellular senescence has been shown to activate inflammasomes via various molecules, including DAMPS and SASP [61]. For example, a senescent gut barrier enables microbial components, such as LPS, to migrate into the circulatory system and activate inflammasomes. It has been established that LPS binds to TLR-4, “priming” the inflammasome, after which a second signal, such as excessive ROS, fully activates these complexes, triggering neuroinflammation. Since S100A9 also attaches to TLR-4, it can prime microglia, predisposing to gray matter volume loss when the second signal becomes available [62,63]. Several studies have found that, like LPS, psychosocial stress can upregulate S100A9, leading to gray matter loss, highlighting the role of stressors in the pathogenesis of SCZ [31,62,63,64] (Figure 2).

### 2.2. AhR and Microglia

**Figure 3:**
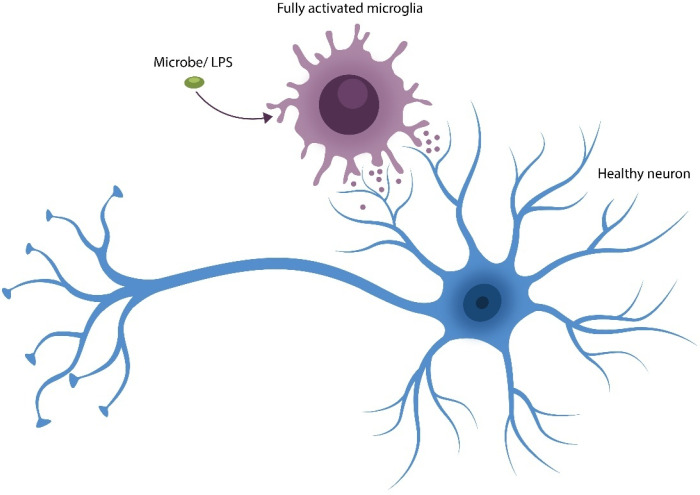
Microglia gone rogue: the elimination of healthy neurons and synapses. Translocated microbes or their components, such as LPS, activate microglial inflammasomes, converting these brain macrophages into a neurotoxic phenotype that can engage in the pathological elimination of viable neurons and synapses, leading to gray matter depletion.

Microglia, the primary brain macrophages, are mobile and vigilant cells that constantly search for, find, and phagocytose molecular debris and dead and damaged cells. Microglia drive synaptic pruning during development and modulate synaptic plasticity in adulthood. The physiological role of microglia, including the elimination of dead or dying neurons, averts inflammation induced by the accumulation of molecular waste. Under pathological circumstances, excessive inflammation can aberrantly activate microglia, leading to the elimination of viable neurons and synapses as documented in SCZ [65] (Figure 3).

AhR mediates the toxic effects of dioxins and other environmental toxicants associated with gray matter volume loss. Along these lines, epidemiological studies have found that prepartum or postpartum exposure to dioxin-like substances can contribute to accelerated gray matter depletion [66,67,68]. Likewise, in elderly individuals, activated AhR has been linked to gray matter atrophy, further connecting this transcription factor to cortical volume reduction [69]. Along these lines, AhR knockout mice showed the preservation of hippocampal volume, directly linking this receptor to gray matter reduction [70]. Other animal studies have implicated activated AhR in neuroinflammation and microglial phagocytosis of healthy neurons and synapses [71]. Furthermore, kynurenine-activated AhR in microglia can convert these immune cells into a neurotoxic phenotype, connecting tryptophan dysmetabolism to gray matter depletion [72,73]. Taken together, these findings indicate that AhR and S100A9 activation in microglia are the likely molecular triggers of SCZ-related gray matter loss [74]. Conversely, inhibiting S100A9 with quinoline-3-carboxamides (paquinimod or laquinimod) may decrease gray matter reduction, pointing to a potential SCZ treatment [75]. In addition, modulating AhR signaling via nutritional ligands such as indole-3-carbinol or flavonoids is currently being explored as a therapeutic option for averting gray matter depletion [76].

## 3. Gray Matter Volume Loss and Schizophrenia Outcome

Neuroimaging studies over the past two decades have shown that the first episode of SCZ is marked by a temporo-parietal gray matter reduction that spreads progressively throughout the brain despite treatment with antipsychotic drugs. This is in line with the observed clinical progression of SCZ. Although the resolution of symptoms and partial recovery are attainable, sustained recovery without relapse, independent living, stable employment, and the ability to raise a family are infrequently seen [77].

Kraepelin conceptualized SCZ as dementia praecox and believed that complete return to the premorbid level of function was rare and unimpressive [78]. At present, this is reflected in the fact that psychiatric state hospitals continue to exist, while public institutions for infectious diseases, such as tuberculosis or leprosy, have been closed for almost a century [79]. This is consistent not only with Kraepelin’s model, but also with Richard Wagner’s study on SCZ outcomes from 1901 to 1995 [80]. This study found that a small number of SCZ patients recover completely and even fewer are capable of maintaining stable employment [80]. Moreover, treatment with antipsychotic drugs (available in the 1950s) has not significantly altered long-term SCZ outcomes. For example:

1. 1901–1920, 20% complete recovery, 4.7% employed;
2. 1921–1940, 12% complete recovery, 11.9% employed;
3. 1941–1955, 23% complete recovery, 4.1% employed;
4. 1956–1975, 20% complete recovery, 5.1% employed;
5. 1976–1995, 20% complete recovery, 6.9% employed.

Furthermore, current epidemiological data shows that 33% of patients with SCZ relapse during the first 12 months after an initial psychotic episode and 26% remain homeless at two-year follow-up, while five years after the first psychotic outbreak, only 10% are employed [81,82,83] (Table 1).

SCZ outcome studies are in line with the progressive gray matter volume loss and limited recovery observed after an initial psychotic episode.

### Phases of Schizophrenia and Gray Matter Depletion

SCZ is a neurodevelopmental disorder believed to originate in utero and progress throughout the entire lives of patients. Genetics and epigenetics were shown to interact, contributing to this syndrome marked by positive and negative symptoms that unfold in four stages.

**Figure 4:**
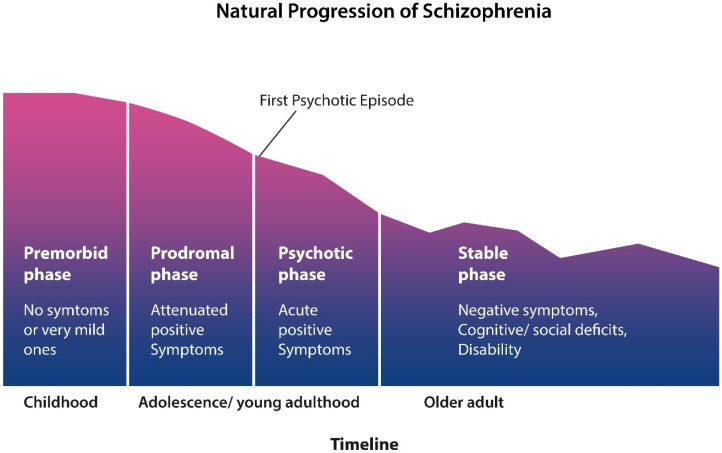
SCZ starts in early childhood with a premorbid phase with few or no clinical symptoms. The prodromal phase, marked by mild, nonspecific symptoms such as insomnia, isolation, and anxiety can last months to years. The third stage, the psychotic phase, is characterized by multiple hospitalizations due to overt positive symptoms and disruptive behavior. Around midlife, the positive symptoms gradually subside and are replaced by negative symptoms and cognitive impairment, resulting in disability (figure adapted from Liberman).

In childhood, there is an asymptomatic phase that may or may not be marked by delayed developmental milestones. Although a small number of studies have shown abnormal neurodevelopment or “pandysmaturation” in infancy, it is generally believed that SCZ cannot be reliably predicted in this phase [84,85]. The premorbid stage is followed by a prodrome that can last from months to years and is marked by mild, but not overt psychotic, symptoms such as insomnia, peculiar beliefs, and internal preoccupation [86]. The psychotic phase is manifested by exacerbations and remissions of psychotic phenomena, such as delusions and hallucinations, often resulting in multiple hospitalizations. The “stable” phase is characterized by negative and cognitive symptoms leading to impairment in almost all activities of daily living, resulting in disability (Figure 4). Throughout the four phases of SCZ, the volume of gray matter continues to decrease, engendering a clinical picture of neurocognitive impairment resembling dementia.

**Table 1:** Stages of schizophrenia with characteristic symptoms.

Stages	Characterization	References
Premorbid	May present with early developmental delays	[87]
Prodromal	Sub-threshold symptoms	[88]
Psychotic	Onset of full psychosis, including hallucinations, delusions, and disorganized speech	[89]
Stable	Cognitive difficulties or social withdrawal	[90]

Several preclinical studies have found a relationship between gray matter depletion and low levels of interleukin-22 (IL-22), especially in the amygdala and cingulate cortex. Under physiological conditions, IL-22 acts as a protective cytokine that promotes tissue repair, while low IL-22 has been linked to the continued reduction of gray matter volume, suggesting a role in SCZ outcome [91].

## 4. IL-22, Gut Permeability and Gray Matter Volume

Under normal circumstances, a limited amount of gut microbes “escape” the GI tract and “train” host immune cells in responding to antigens. However, a massive migration of intestinal microorganisms or toxins into the circulatory system is pathological, as peripheral immunity is intolerant of these “intruders” and responds by robust inflammation. In the brain, gut microbes and their components may trigger psychosis via several mechanisms, including neuroinflammation or AhR activation with premature cellular senescence that may lead to aberrant microglial activation and gray matter loss [92,93]. For example, *Escherichia coli* (*E. coli*) is an established “microbial migrant” from the GI tract into systemic circulation known for triggering pathology by accumulating in various tissues, including the urinary bladder, the brain, or atherosclerotic plaques [94,95,96]. For example, an *E. coli* outbreak in 2011 in northern Germany was associated with some cases of new-onset psychosis, connecting neuropsychiatric illness to this bacterium previously linked to SCZ [97,98].

During the human immunodeficiency virus (HIV) epidemic of the 1980s, it was found that this pathogen depletes interleukin-22 (IL-22), generating a high translocation of gut microbes and LPS into systemic circulation, causing pathology [99,100]. Moreover, compared to the general population, individuals with HIV are more likely to develop new-onset psychosis, suggesting a role of LPS and/or gut microbes in the pathogenesis of this condition [101,102].

IL-22 is a member of IL-10 family and is released by several types of lymphocytes, including T helper (Th) 17 cells, γδ T cells, NKCs, and innate lymphoid cells (ILCs). The receptor for IL-22 is a dimeric protein that also binds IL-10. This receptor regulates the JAK/STAT pathway, an innate cellular antiviral and antimicrobial system [103]. In IECs, the JAK/STAT pathway regulates barrier function, including mucus formation and antimicrobial peptides, linking deficient IL-22 to translocation disorders [104]. In addition, as IL-22 was shown to possess neuroprotective properties, its dysfunction has been associated with SCZ [105].

Several preclinical studies have reported that IL-22 protects gray matter by preventing neuronal death. For example, IL-22-deficient mice exhibit more gray matter damage and axonal loss compared to wild-type rodents. Indeed, in animal models of ischemia, Alzheimer’s disease (AD), and multiple sclerosis (MS), the administration of recombinant IL-22 preserved the loss of neurons and gray matter volume, indicating a potential SCZ treatment [106,107,108,109]. In this regard, recombinant IL-22 has recently been patented for use in SCZ (publication number 20240277809). Moreover, IL-22 was demonstrated to augment long-term potentiation and memory by promoting adult neurogenesis [110]. Interestingly, IL-22 can enhance the restorative inflammation needed for wound healing and protection against tumorigenesis, while at the same time deterring pathological inflammation [111]. Due to its anti-apoptotic action, IL-22 promotes tissue repair and homeostasis, but may fuel tumor progression [112]. However, recombinant human IL-22 administered in therapeutic, controlled settings is generally well tolerated [113].

Taken together, current literature highlights the fact that IL-22 protects gray matter in animal models of CNS disease. IL-22-induced inflammation is adaptive in nature and promotes healing. This has contributed to the study of recombinant IL-22 as a potential therapy for SCZ.

## 5. The Microbiome Antipsychotic System

Phenazines are microbial metabolites and AhR ligands that exert antibacterial, anticancer, and antipsychotic properties [114]. Microbial phenazines, generated by several soil, seawater, and gut microorganisms including Streptococcus species and Pseudomonas aeruginosa, were demonstrated to exhibit neuroprotective properties. The structural similarities of these molecules with synthetic antipsychotic drugs, phenothiazines, suggest that humans may possess an inbuilt antipsychotic system akin to the analgesic pro-opiomelanocortin (POMC) pathway.

Phenazines possess antioxidant properties and may protect neuronal cells against excitotoxicity. Furthermore, both microbial phenazines and phenothiazines are AhR ligands, further implicating this receptor in CNS health and disease [115]. Moreover, the synthetic phenazines pontemazine A and B, derived from *Streptomyces* sp. *UT1123*, were shown to possess neuroprotective properties against glutamate cytotoxicity, suggesting new treatment options for SCZ [116].

## 6. Potential Interventions for Barrier Dysfunction

Considering the data presented here, several interventions for lowering gray matter depletion may be applicable to SCZ. Unlike antipsychotic drugs that act in a symptomatic manner, these strategies may address the root cause of underlying pathology by restoring TJs or decreasing the consequences of cellular senescence, such as SASP ( Table 2 ).

**Table 2:** Potential interventions.

Agent	Mechanism	References
Cysteine	SCZ augmentation	[117]
Glutamine	Barrier enhancement	[118]
*Indigo Naturalis*	Barrier enhancement	[119]
Alstonine	SCZ augmentation	[120]
Quercetin	SCZ augmentation	[121]
Curcumin	SCZ augmentation	[122]
Luteolin	SCZ augmentation	[123]
Vagal nerve stimulation	Barrier enhancement	[124]
Rituximab	Barrier enhancement	[125]
Butyrate	SCZ augmentation	[126]
Laquinimod	Barrier augmentation	[127]

### 6.1. Amino Acids

#### 6.1.1. Cysteine

Cysteine is a non-essential amino acid that plays a major role in protein synthesis and the generation of antioxidant systems in cells. For example, a functional cysteine/glutathione (GSH)/glutathione peroxidase 4 (GPX4) axis is crucial for preventing neuronal demise by ferroptosis, a programmed cell death modality, recently documented in SCZ [128]. In the gut, cysteine prevents the ferroptosis of IECs, a pathology demonstrated in IBD, a condition marked by the excessive translocation of gut microbes or their molecules into systemic circulation [129].

#### 6.1.2. Glutamine

Several studies have reported that amino acids, including glutamine and L-glutamate, augment TJs and restore the integrity of intestinal mucosa after LPS damage [130,131,132]. As glutamine is the principal metabolite of synaptic glutamate and patients with chronic SCZ exhibit an altered glutamine/glutamate ratio, glutamine supplementation may add nutritional benefits to conventional antipsychotic treatment [133,134]. Interestingly, AhR has been implicated in glutamate and aspartate transport, further linking this transcription factor to SCZ [135].

### 6.2. Phytotherapy

#### 6.2.1. *Indigo Naturalis* (*IN*)

*Indigo naturalis* (*IN*) is a traditional Chinese medicinal plant derived from *Strobilanthes formosana* (*Acanthaceae*) that can repair the gut barrier via its AhR-binding indole alkaloids that promote IL-22 synthesis [136]. In addition, a recent study found that IN targets GSK-3β, an IL-22-altering pathway implicated in SCZ and IBD [137,138].

#### 6.2.2. *Catharanthus roseus*

Alstonine is an indole alkaloid found abundantly in some plants, including *Catharanthus roseus*, a tropical plant native to Madagascar. Alstonine was demonstrated to possess antipsychotic properties comparable to those of clozapine and has been used in Africa as a natural remedy for the treatment of mental illness [139]. As an indole derivative, alstonine is likely an AhR ligand that protects the gut barrier function, therefore preventing the translocation of microbes and their components [140].

### 6.3. Polyphenols

#### Quercetin

Quercetin is a natural flavonoid and AhR ligand that enhances TJs, preventing microbial translocation that may benefit patients with IBD [141]. Indeed, several studies have found that quercetin facilitates the expression of claudin-1, claudin-4, zonulin-2, and occludin, proteins capable of restoring gut barrier function [142,143]. In the CNS, quercetin acts as a negative allosteric modulator of GABAA receptors (GABAA-Rs), explaining its anti-inflammatory and neuroprotective role in gray matter preservation [144,145,146,147]. Moreover, quercetin exerts antipsychotic activity, suggesting usefulness as a complementary SCZ therapy [148,149].

### 6.4. Curcumin

Curcumin is an AhR modulator with anti-inflammatory properties that enhances the intestinal barrier by promoting beneficial gut microbes [150]. In SCZ, curcumin promotes neuroplasticity and has been found to be beneficial against negative symptoms [151,152].

### 6.5. Luteolin

Luteolin is an AhR ligand that reduces microglial inflammation and gray matter depletion [153]. Luteolin is currently in clinical trials for the treatment of SCZ (NCT05204407).

### 6.6. Vagal Nerve Stimulation (VNS)

In 2005, the Food and Drug Administration (FDA) approved vagal nerve stimulation (VNS) for the treatment of refractory major depressive disorder (MDD). However, the action mechanism of this modality has not been completely elucidated until recently. Several studies have shown that VNS enhances TJs, preventing the translocation of microbes and their components from the intestine into systemic circulation [124,151]. The noninvasive VNS counterpart, transcutaneous auricular vagal nerve stimulation (taVNS), currently used for seizure disorder and migraine headaches, was found to protect the intestinal barrier via the parasympathetic nicotinic enhancement of occludin and zonulin-1 [152,153]. This modality may provide therapeutic advantages in SCZ by preventing translocation and aberrant microglial activation [154,155].

### 6.7. Other Interventions

Rituximab, a monoclonal antibody developed for autoimmune disorders, was found to improve the clearance of damaged cells, including IECs, thus restoring physiological gut permeability and lowering microbial and LPS translocation [156]. Recently, rituximab has been studied as a potential treatment for resistant SCZ [157].

Butyrate is a microbiome-generated short-chain fatty acid (SCFA) that plays a critical role in maintaining the integrity of the gut and BBB. As previous studies found that SCZ patients exhibited decreased abundance of gut butyrate-producing bacteria, it was suggested that supplementation with this SCFA may be therapeutic [158]. Indeed, butyrate is an AhR ligand shown to restore TJs, averting microbial and LPS translocation outside the GI tract [159,160].

Paquinimod and laquinimod are AhR activators belonging to the class of quinoline-3-carboxamides. Experimental data suggests that these compounds enhance gut and BBB function, averting translocation [161,162]. These anti-MS drugs may be beneficial in SCZ as they reduce microglial activation and gray matter depletion [162,163].

### 6.8. Limitations and Further Directions

This review has the following limitations:

1. AhR variants were documented in ASD but, to our knowledge, there are no SCZ studies on this matter.
2. The treatments proposed here, such as vagal nerve stimulation, have not been studied in SCZ; however, the FDA approval of the cholinergic drug Cobenfy suggests that other cholinergic agents and procedures might be helpful. For example, cholinesterase inhibitors (ChEIs) have been previously explored in SCZ with negative symptoms.
3. Although increased inflammatory cytokines and inflammasome activation were demonstrated in humans with SCZ, administering LPS to human subjects is unethical and there are no meaningful studies in this matter.
4. Higher numbers of antibodies against *E. coli* were demonstrated in small studies on humans with SCZ (suggesting microbial translocation); however, larger studies are needed to demonstrate the role of the gut–brain axis in this condition.
5. Although primary central immune dysregulation could explain neuroinflammation in SCZ, elevated levels of LPS and related biomarkers in the peripheral blood of many patients suggest that gut permeability issues are likely responsible for the systemic inflammation that affects the brain.
6. The findings proposed in this paper are intended to generate testable hypotheses rather than to represent a confirmed pathogenic pathway.

## 7. Conclusions

Various SCZ hypotheses propose that this condition could be caused by disrupted brain development or neurotransmitter imbalance (primarily dopamine, but also glutamate/GABA and others) that may be triggered by genetic or environmental factors. Newer theories explore immune and synaptic dysfunction to explain complex symptoms such as hallucinations, delusions, and cognitive issues. The microbial model is an old paradigm promoted by Kraepelin that had been forgotten until the discovery of the microbiome. The connection between *E. coli* and SCZ is well known to practicing psychiatrists who often witness psychotic decompensation after urinary tract infections or pneumonia.

Reduced brain gray matter volume and enlarged lateral ventricles are among the earliest and most consistent morphometric findings in SCZ. Genetic or neurodevelopmental factors could be responsible for gray matter volume reduction, but some genes, like DISC1 (Disrupted in Schizophrenia 1) and the Dopamine D2 receptor, have been implicated in both SCZ and IBD (a condition marked by the extensive migration of bacteria outside the GI tract). In addition, prenatal or neonatal exposure to LPS, an endotoxin from Gram-negative bacteria, is strongly linked to neurodevelopmental disabilities, indicating that genetic, developmental, and microbial factors are not mutually exclusive but highly intertwined.

Like the gut barrier, microglia express abundant AhR, a receptor for several SCZ-relevant neurotransmitters, psychotropic drugs, and microbial molecules. Microglia activated by bacteria or their components can engage in the aberrant phagocytosis of healthy brain tissue, leading to reduced gray matter. This may reflect clinically in progressive deficits, disability, and less-than-optimal sustained recovery.

Restoring gut barrier integrity and lowering the influx of immunogenic molecules from the GI tract into the circulatory system can avert gray matter loss and enhance SCZ recovery.

## Data Availability

No new data were created or analyzed in this study.

## Abbreviations

SCZ	schizophrenia
PET	positron emission tomography
MRI	magnetic resonance imaging
SMI	severe mental illness
sCD14	soluble CD14
IBD	inflammatory bowel disease
LPS	lipopolysaccharide
HIV	human immunodeficiency virus
BBB	blood–brain barrier
IEC	intestinal epithelial cell
DAMP	lipopolysaccharide
SASP	senescence-associated secretory phenotype
AhR	aryl hydrocarbon receptor
TMAO	trimethylamine-N-oxide
UVR	ultraviolet radiation
PAH	polycyclic aromatic hydrocarbons
GPX4	glutathione peroxidase 4

## References

[1] Thompson P.M., Vidal C., Giedd J.N., Gochman P., Blumenthal J., Nicolson R., Toga A.W., Rapoport J.L. (2001). Mapping adolescent brain change reveals dynamic wave of accelerated gray matter loss in very early-onset schizophrenia. Proc. Natl. Acad. Sci. USA.

[2] Wang X.-L., Li L. (2021). Microglia Regulate Neuronal Circuits in Homeostatic and High-Fat Diet-Induced Inflammatory Conditions. Front. Cell. Neurosci..

[3] Lindhout I.A., Murray T.E., Richards C.M., Klegeris A. (2021). Potential neurotoxic activity of diverse molecules released by microglia. Neurochem. Int..

[4] Severance E.G., Gressitt K.L., Stallings C.R., Origoni A.E., Khushalani S., Leweke F.M., Dickerson F.B., Yolken R.H. (2013). Discordant patterns of bacterial translocation markers and implications for innate immune imbalances in schizophrenia. Schizophr. Res..

[5] Wang C., Zhang T., He L., Fu J.-Y., Deng H.-X., Xue X.-L., Chen B.-T. (2021). Bacterial Translocation Associates with Aggression in Schizophrenia Inpatients. Front. Syst. Neurosci..

[6] Sung K., Zhang B., Wang H.E., Bai Y., Tsai S., Su T., Chen T., Hou M., Lu C., Wang Y. (2022). Schizophrenia and risk of new-onset inflammatory bowel disease: A nationwide longitudinal study. Aliment. Pharmacol. Ther..

[7] Massironi S., Pigoni A., Vegni E.A.M., Keefer L., Dubinsky M.C., Brambilla P., Delvecchio G., Danese S. (2024). The Burden of Psychiatric Manifestations in Inflammatory Bowel Diseases: A Systematic Review with Meta-analysis. Inflamm. Bowel Dis..

[8] Bernstein C.N., Hitchon C.A., Walld R., Bolton J.M., Sareen J., Walker J.R., Graff L.A., Patten S.B., Singer A., Lix L.M. (2018). Increased Burden of Psychiatric Disorders in Inflammatory Bowel Disease. Inflamm. Bowel Dis..

[9] Ludvigsson J.F., Olén O., Larsson H., Halfvarson J., Almqvist C., Lichtenstein P., Butwicka A. (2021). Association Between Inflammatory Bowel Disease and Psychiatric Morbidity and Suicide: A Swedish Nationwide Population-Based Cohort Study with Sibling Comparisons. J. Crohn’s Colitis.

[10] Harris M.J., Jeste D.V., Gleghorn A., Sewell D.D. (1991). New-onset psychosis in HIV-infected patients. J. Clin. Psychiatry.

[11] De Ronchi D., Faranca I., Forti P., Ravaglia G., Borderi M., Manfredi R., Volterra V. (2000). Development of Acute Psychotic Disorders and HIV-1 Infection. Int. J. Psychiatry Med..

[12] Chhagan U., Ntlantsana V., Tomita A., Naidu T., Chiliza B., Paruk S. (2021). Investigating the impact of HIV on patients with first episode psychosis: A study protocol for a longitudinal cohort study. BMJ Open.

[13] Hou J.-J., Ma A.-H., Qin Y.-H. (2023). Activation of the aryl hydrocarbon receptor in inflammatory bowel disease: Insights from gut microbiota. Front. Cell. Infect. Microbiol..

[14] Moutusy S.I., Ohsako S. (2024). Gut Microbiome-Related Anti-Inflammatory Effects of Aryl Hydrocarbon Receptor Activation on Inflammatory Bowel Disease. Int. J. Mol. Sci..

[15] Zhou Y.-H., Sun L., Chen J., Sun W.-W., Ma L., Han Y., Jin X., Zhao Q.-X., Li T., Lu H. (2019). Tryptophan Metabolism Activates Aryl Hydrocarbon Receptor-Mediated Pathway to Promote HIV-1 Infection and Reactivation. mBio.

[16] Lawrence B.P., Vorderstrasse B.A. (2013). New insights into the aryl hydrocarbon receptor as a modulator of host responses to infection. Semin. Immunopathol..

[17] Yu M., Wang Q., Ma Y., Li L., Yu K., Zhang Z., Chen G., Li X., Xiao W., Xu P. (2018). Aryl Hydrocarbon Receptor Activation Modulates Intestinal Epithelial Barrier Function by Maintaining Tight Junction Integrity. Int. J. Biol. Sci..

[18] Wang X., Hawkins B.T., Miller D.S. (2010). Aryl hydrocarbon receptor-mediated up-regulation of ATP-driven xenobiotic efflux transporters at the blood-brain barrier. FASEB J..

[19] Koizumi M., Tatebe J., Watanabe I., Yamazaki J., Ikeda T., Morita T. (2014). Aryl Hydrocarbon Receptor Mediates Indoxyl Sulfate-Induced Cellular Senescence in Human Umbilical Vein Endothelial Cells. J. Atheroscler. Thromb..

[20] Salminen A. (2022). Aryl hydrocarbon receptor (AhR) reveals evidence of antagonistic pleiotropy in the regulation of the aging process. Cell. Mol. Life Sci..

[21] Nacarino-Palma A., Rico-Leo E.M., Campisi J., Ramanathan A., González-Rico F.J., Rejano-Gordillo C.M., Ordiales-Talavero A., Merino J.M., Fernández-Salguero P.M. (2022). Aryl hydrocarbon receptor blocks aging-induced senescence in the liver and fibroblast cells. Aging.

[22] Sfera A., Thomas K.A., Anton J. (2024). Cytokines and Madness: A Unifying Hypothesis of Schizophrenia Involving Interleukin-22. Int. J. Mol. Sci..

[23] Sfera A. (2023). Six Decades of Dopamine Hypothesis: Is Aryl Hydrocarbon Receptor the New D2?. Reports.

[24] Shi L., Zhao Y., Fei C., Guo J., Jia Y., Wu D., Wu L., Chang C. (2019). Cellular senescence induced by S100A9 in mesenchymal stromal cells through NLRP3 inflammasome activation. Aging.

[25] Pampuscenko K., Jankeviciute S., Morkuniene R., Sulskis D., Smirnovas V., Brown G.C., Borutaite V. (2025). S100A9 protein activates microglia and stimulates phagocytosis, resulting in synaptic and neuronal loss. Neurobiol. Dis..

[26] Lv J., Wang X., Qin W. (2025). Integrated bioinformatics and machine learning identify S100A9 and VGLL1 as hub genes for schizophrenia. Front. Psychiatry.

[27] Ma L., Sun P., Zhang J.-C., Zhang Q., Yao S.-L. (2017). Proinflammatory effects of S100A8/A9 via TLR4 and RAGE signaling pathways in BV-2 microglial cells. Int. J. Mol. Med..

[28] Zhang X., Sun D., Zhou X., Zhang C., Yin Q., Chen L., Tang Y., Liu Y., Morozova-Roche L.A. (2023). Proinflammatory S100A9 stimulates TLR4/NF-κB signaling pathways causing enhanced phagocytic capacity of microglial cells. Immunol. Lett..

[29] Butler C.A., Popescu A.S., Kitchener E.J.A., Allendorf D.H., Puigdellívol M., Brown G.C. (2021). Microglial phagocytosis of neurons in neurodegeneration, and its regulation. J. Neurochem..

[30] Garcia V., Perera Y.R., Chazin W.J. (2022). A Structural Perspective on Calprotectin as a Ligand of Receptors Mediating Inflammation and Potential Drug Target. Biomolecules.

[31] Swindell W.R., Johnston A., Xing X., Little A., Robichaud P., Voorhees J.J., Fisher G., Gudjonsson J.E. (2013). Robust shifts in S100a9 expression with aging: A novel mechanism for chronic inflammation. Sci. Rep..

[32] Guan X., Zha L., Zhu X., Rao X., Huang X., Xiong Y., Guo Y., Zhang M., Zhou D., Tu Q. (2025). Mechanism of action and therapeutic potential of S100A8/A9 in neuroinflammation and cognitive impairment: From molecular target to clinical application (Review). Int. J. Mol. Med..

[33] Averina O.V., Poluektova E.U., Zorkina Y.A., Kovtun A.S., Danilenko V.N. (2024). Human Gut Microbiota for Diagnosis and Treatment of Depression. Int. J. Mol. Sci..

[34] Juricek L., Coumoul X. (2018). The Aryl Hydrocarbon Receptor and the Nervous System. Int. J. Mol. Sci..

[35] Sharma R. (2022). Emerging Interrelationship Between the Gut Microbiome and Cellular Senescence in the Context of Aging and Disease: Perspectives and Therapeutic Opportunities. Probiot. Antimicrob. Proteins.

[36] Frey N., Venturelli S., Zender L., Bitzer M. (2017). Cellular senescence in gastrointestinal diseases: From pathogenesis to therapeutics. Nat. Rev. Gastroenterol. Hepatol..

[37] Koren T., Yifa R., Amer M., Krot M., Boshnak N., Ben-Shaanan T.L., Azulay-Debby H., Zalayat I., Avishai E., Hajjo H. (2021). Insular cortex neurons encode and retrieve specific immune responses. Cell.

[38] Gong Y., Chen A., Zhang G., Shen Q., Zou L., Li J., Miao Y.-B., Liu W. (2023). Cracking Brain Diseases from Gut Microbes-Mediated Metabolites for Precise Treatment. Int. J. Biol. Sci..

[39] Li D., Ke Y., Zhan R., Liu C., Zhao M., Zeng A., Shi X., Ji L., Cheng S., Pan B. (2018). Trimethylamine-*N*-oxide promotes brain aging and cognitive impairment in mice. Aging Cell.

[40] Bonaparte J., Cook M., Dixon S., Wright C., Hill W., Wang G. (2023). Kynurenine Metabolites Induce Microglial Cell Senescence and Stimulate Neuroinflammation. Innov. Aging.

[41] Pathak S., Nadar R., Kim S., Liu K., Govindarajulu M., Cook P., Alexander C.S.W., Dhanasekaran M., Moore T. (2024). The Influence of Kynurenine Metabolites on Neurodegenerative Pathologies. Int. J. Mol. Sci..

[42] Kim H., Lee E., Park M., Min K., Diep Y.N., Kim J., Ahn H., Lee E., Kim S., Kim Y. (2024). Microbiome-derived indole-3-lactic acid reduces amyloidopathy through aryl-hydrocarbon receptor activation. Brain Behav. Immun..

[43] Wei L., Yang X., Wang J., Wang Z., Wang Q., Ding Y., Yu A. (2023). H3K18 lactylation of senescent microglia potentiates brain aging and Alzheimer’s disease through the NFκB signaling pathway. J. Neuroinflamm..

[44] Ng P.Y., McNeely T.L., Baker D.J. (2021). Untangling senescent and damage-associated microglia in the aging and diseased brain. FEBS J..

[45] Tay T.L., Béchade C., D’Andrea I., St-Pierre M.-K., Henry M.S., Roumier A., Tremblay M.-E. (2018). Microglia Gone Rogue: Impacts on Psychiatric Disorders across the Lifespan. Front. Mol. Neurosci..

[46] Zhu H., Guan A., Liu J., Peng L., Zhang Z., Wang S. (2023). Noteworthy perspectives on microglia in neuropsychiatric disorders. J. Neuroinflamm..

[47] Galle E., Wong C.-W., Ghosh A., Desgeorges T., Melrose K., Hinte L.C., Castellano-Castillo D., Engl M., de Sousa J.A., Ruiz-Ojeda F.J. (2022). H3K18 lactylation marks tissue-specific active enhancers. Genome Biol..

[48] Föcking M., Doyle B., Munawar N., Dillon E.T., Cotter D., Cagney G. (2019). Epigenetic Factors in Schizophrenia: Mechanisms and Experimental Approaches. Complex Psychiatry.

[49] Wiley C.D., Campisi J. (2016). From Ancient Pathways to Aging Cells—Connecting Metabolism and Cellular Senescence. Cell Metab..

[50] Chen A.-N., Luo Y., Yang Y.-H., Fu J.-T., Geng X.-M., Shi J.-P., Yang J. (2021). Lactylation, a Novel Metabolic Reprogramming Code: Current Status and Prospects. Front. Immunol..

[51] Yuan J.-Q., Wang S.-M., Guo L. (2023). S100A9 promotes glycolytic activity in HER2-positive breast cancer to induce immunosuppression in the tumour microenvironment. Heliyon.

[52] Wang Y., Ni T., Zhang Q., Xu Z., Zhu Z., Xie J., Yi M., Tu L., Cheng Z., Gao Y. (2025). AhR deficiency exacerbates inflammation in diabetic wounds via impaired mitophagy and cGAS-STING-NLRP3 activation: Therapeutic potential of hydrogels loaded with FICZ. Mater. Today Bio.

[53] Dahlem C., Kado S.Y., He Y., Bein K., Wu D., Haarmann-Stemmann T., Kado N.Y., Vogel C.F.A. (2020). AHR Signaling Interacting with Nutritional Factors Regulating the Expression of Markers in Vascular Inflammation and Atherogenesis. Int. J. Mol. Sci..

[54] Memari B., Nguyen-Yamamoto L., Salehi-Tabar R., Zago M., Fritz J.H., Baglole C.J., Goltzman D., White J.H. (2019). Endocrine aryl hydrocarbon receptor signaling is induced by moderate cutaneous exposure to ultraviolet light. Sci. Rep..

[55] Davis G.E., Davis M.J., Lowell W.E. (2022). The effect of ultraviolet radiation on the incidence and severity of major mental illness using birth month, birth year, and sunspot data. Heliyon.

[56] Yi W., Cheng J., Song J., Pan R., Liang Y., Sun X., Li Y., Wu Y., Yan S., Jin X. (2023). Associations of polycyclic aromatic hydrocarbons, water-soluble ions and metals in PM2.5 with liver function: Evidence from schizophrenia cohort. Sci. Total. Environ..

[57] Liu X., Wang J., Jin J., Hu Q., Zhao T., Wang J., Gao J., Man J. (2024). S100A9 deletion in microglia/macrophages ameliorates brain injury through the STAT6/PPARγ pathway in ischemic stroke. CNS Neurosci. Ther..

[58] Shirley S.H., von Maltzan K., Robbins P.O., Kusewitt D.F. (2014). Melanocyte and Melanoma Cell Activation by Calprotectin. J. Ski. Cancer.

[59] Gober R., Dallmeier J., Davis D., Brzostowicki D., de Rivero Vaccari J.P., Cyr B., Barreda A., Sun X., Gultekin S.H., Garamszegi S. (2024). Increased inflammasome protein expression identified in microglia from postmortem brains with schizophrenia. J. Neuropathol. Exp. Neurol..

[60] Popovački E.Š., Vogrinc D., Fuller H.R., Horvat L.L., Mayer D., Kopić J., Pintarić K., Leko M.B., Pravica M., Krsnik Ž. (2024). Increased NLRP1 mRNA and Protein Expression Suggests Inflammasome Activation in the Dorsolateral Prefrontal and Medial Orbitofrontal Cortex in Schizophrenia. Biomolecules.

[61] Mittal R., Saavedra D., Mittal M., Lemos J.R.N., Hirani K. (2025). Inflammasome activation and accelerated immune aging in autoimmune disorders. Front. Aging.

[62] Jonasson L., Larsen H.G., Lundberg A.K., Gullstrand B., Bengtsson A.A., Schiopu A. (2017). Stress-induced release of the S100A8/A9 alarmin is elevated in coronary artery disease patients with impaired cortisol response. Sci. Rep..

[63] Iwata M., Ota K.T., Duman R.S. (2013). The inflammasome: Pathways linking psychological stress, depression, and systemic illnesses. Brain Behav. Immun..

[64] Ozeki A., Oogaki Y., Henmi Y., Karasawa T., Takahashi M., Takahashi H., Ohkuchi A., Shirasuna K. (2021). Elevated S100A9 in preeclampsia induces soluble endoglin and IL-1β secretion and hypertension via the NLRP3 inflammasome. J. Hypertens..

[65] Hartmann S.-M., Heider J., Wüst R., Fallgatter A.J., Volkmer H. (2024). Microglia-neuron interactions in schizophrenia. Front. Cell. Neurosci..

[66] Yamazaki K., Itoh S., Ikeda-Araki A., Miyashita C., Hori T., Hachiya N., Kishi R. (2022). Association of prenatal exposure to dioxin-like compounds, polychlorinated biphenyl, and methylmercury with event-related brain potentials in school-aged children: The Hokkaido study. NeuroToxicology.

[67] de Chastelaine M., Srokova S., Hou M., Kidwai A., Kafafi S.S., Racenstein M.L., Rugg M.D. (2023). Cortical thickness, gray matter volume, and cognitive performance: A crosssectional study of the moderating effects of age on their interrelationships. Cereb. Cortex.

[68] Vreugdenhil H.J., Lanting C.I., Mulder P.G., Boersma E., Weisglas-Kuperus N. (2002). Effects of prenatal PCB and dioxin background exposure on cognitive and motor abilities in Dutch children at school age. J. Pediatr..

[69] Ojo E.S., Tischkau S.A. (2021). The Role of AhR in the Hallmarks of Brain Aging: Friend and Foe. Cells.

[70] Latchney S.E., Hein A.M., O’Banion M.K., DiCicco-Bloom E., Opanashuk L.A. (2012). Deletion or activation of the aryl hydrocarbon receptor alters adult hippocampal neurogenesis and contextual fear memory. J. Neurochem..

[71] Zhou Y., Zhao W.-J., Quan W., Qiao C.-M., Cui C., Hong H., Shi Y., Niu G.-Y., Zhao L.-P., Shen Y.-Q. (2021). Dynamic changes of activated AHR in microglia and astrocytes in the substantia nigra-striatum system in an MPTP-induced Parkinson’s disease mouse model. Brain Res. Bull..

[72] Garrison A.M., Parrott J.M., Tuñon A., Delgado J., Redus L., O’connor J.C. (2018). Kynurenine pathway metabolic balance influences microglia activity: Targeting kynurenine monooxygenase to dampen neuroinflammation. Psychoneuroendocrinology.

[73] de la Flor M.A., O’Connor J.C. (2025). Losing the Filter: How Kynurenine Pathway Dysregulation Impairs Habituation. Cells.

[74] Wu M., Xu L., Wang Y., Zhou N., Zhen F., Zhang Y., Qu X., Fan H., Liu S., Chen Y. (2018). S100A8/A9 induces microglia activation and promotes the apoptosis of oligodendrocyte precursor cells by activating the NF-κB signaling pathway. Brain Res. Bull..

[75] Sharma G., Malik A., Tripathi S., Deshmukh V., Patil A. (2024). Gene expression analysis of Schizophrenia. Bioinformation.

[76] Ayaz M., Sadiq A., Junaid M., Ullah F., Ovais M., Ullah I., Ahmed J., Shahid M. (2019). Flavonoids as Prospective Neuroprotectants and Their Therapeutic Propensity in Aging Associated Neurological Disorders. Front. Aging Neurosci..

[77] Insel T.R. (2010). Rethinking schizophrenia. Nature.

[78] Kraepelin E. (1896). Psychiatrie: Ein Lehrbuch fur Studirende und Aerzte. Fünfte, Vollständig Umgearbeitete Auflage.

[79] Zipursky R.B. (2014). Why Are the Outcomes in Patients with Schizophrenia So Poor?. J. Clin. Psychiatry.

[80] Pfohl S., Warner R. (1987). Recovery from Schizophrenia: Psychiatry and Political Economy. Contemp. Sociol. A J. Rev..

[81] Holm M., Taipale H., Tanskanen A., Tiihonen J., Mitterdorfer-Rutz E. (2020). Employment among people with schizophrenia or bipolar disorder: A population-based study using nationwide registers. Acta Psychiatr. Scand..

[82] Lévesque I.S., Abdel-Baki A. (2019). Homeless youth with first-episode psychosis: A 2-year outcome study. Schizophr. Res..

[83] Davidson L., Schmutte T., Dinzeo T., Andres-Hyman R. (2007). Remission and Recovery in Schizophrenia: Practitioner and Patient Perspectives. Schizophr. Bull..

[84] Fish B., Kendler K.S. (2005). Abnormal Infant Neurodevelopment Predicts Schizophrenia Spectrum Disorders. J. Child Adolesc. Psychopharmacol..

[85] Gilmore J.H., Kang C., Evans D.D., Wolfe H.M., Smith J.K., Lieberman J.A., Lin W., Hamer R.M., Styner M., Gerig G. (2010). Prenatal and Neonatal Brain Structure and White Matter Maturation in Children at High Risk for Schizophrenia. Am. J. Psychiatry.

[86] Klosterkotter J., Schultze-Lutter F., Bechdolf A., Ruhrmann S. (2011). Prediction and prevention of schizophrenia: What has been achieved and where to go next?. World Psychiatry.

[87] Sørensen H.J., Mortensen E.L., Schiffman J., Reinisch J.M., Maeda J., Mednick S.A. (2010). Early developmental milestones and risk of schizophrenia: A 45-year follow-up of the Copenhagen Perinatal Cohort. Schizophr Res..

[88] de Filippis R., Carbone E.A., Rania M., Aloi M., Segura-Garcia C., De Fazio P. (2024). Applying a clinical staging model in patients affected by schizophrenia spectrum disorder. Front Psychiatry.

[89] Tandon R., Nasrallah H.A., Keshavan M.S. (2009). Schizophrenia, “just the facts” 4. Clinical features and conceptualization. Schizophr. Res..

[90] Lewis D.A., Lieberman J.A. (2000). Catching up on schizophrenia: Natural history and neurobiology. Neuron.

[91] Chen J., Yan Y., Yuan F., Cao J., Li S., Eickhoff S.B., Zhang J. (2019). Brain grey matter volume reduction and anxiety-like behavior in lipopolysaccharide-induced chronic pulmonary inflammation rats: A structural MRI study with histological validation. Brain. Behav. Immun..

[92] Franchi L., Muñoz-Planillo R., Núñez G. (2012). Sensing and reacting to microbes through the inflammasomes. Nat. Immunol..

[93] Fernández-Arjona M.d.M., Grondona J.M., Fernández-Llebrez P., López-Ávalos M.D. (2019). Microglial activation by microbial neuraminidase through TLR2 and TLR4 receptors. J. Neuroinflamm..

[94] Carnevale R., Nocella C., Petrozza V., Cammisotto V., Pacini L., Sorrentino V., Martinelli O., Irace L., Sciarretta S., Frati G. (2018). Localization of lipopolysaccharide from *Escherichia coli* into human atherosclerotic plaque. Sci. Rep..

[95] Salazar A.M., Neugent M.L., De Nisco N.J., Mysorekar I.U. (2022). Gut-bladder axis enters the stage: Implication for recurrent urinary tract infections. Cell Host Microbe.

[96] Zhao Y., Cong L., Lukiw W.J. (2017). Lipopolysaccharide (LPS) Accumulates in Neocortical Neurons of Alzheimer’s Disease (AD) Brain and Impairs Transcription in Human Neuronal-Glial Primary Co-cultures. Front. Aging Neurosci..

[97] Kleimann A., Toto S., Eberlein C.K., Kielstein J.T., Bleich S., Frieling H., Sieberer M. (2014). Psychiatric Symptoms in Patients with Shiga Toxin-Producing *E. coli* O104:H4 Induced Haemolytic-Uraemic Syndrome. PLoS ONE.

[98] Wiwanitkit V. (2012). Psychosis and *E. coli* Infection: A Forgotten Issue. Indian J. Psychol. Med..

[99] Sandler N.G., Douek D.C. (2012). Microbial translocation in HIV infection: Causes, consequences and treatment opportunities. Nat. Rev. Microbiol..

[100] Kim C.J., Nazli A., Rojas O.L., Chege D., Alidina Z., Huibner S., Mujib S., Benko E., Kovacs C., Shin L.Y.Y. (2012). A role for mucosal IL-22 production and Th22 cells in HIV-associated mucosal immunopathogenesis. Mucosal Immunol..

[101] Nakasujja N., Musisi S., Agren H., Katabira E., Allebeck P. (2024). Psychotic disorders in HIV-positive versus HIV-negative patients: Comparative study of clinical characteristics. BJPsych Open..

[102] Alciati A., Fusi A., Monforte A.D., Coen M., Ferri A., Mellado C. (2001). New-onset delusions and hallucinations in patients infected with HIV. J. Psychiatry Neurosci..

[103] Ezeonwumelu I.J., Garcia-Vidal E., Ballana E. (2021). JAK-STAT Pathway: A Novel Target to Tackle Viral Infections. Viruses.

[104] Keir M.E., Yi T., Lu T.T., Ghilardi N. (2020). The role of IL-22 in intestinal health and disease. J. Exp. Med..

[105] Subbanna M., Shivakumar V., Talukdar P.M., Narayanaswamy J.C., Venugopal D., Berk M., Varambally S., Venkatasubramanian G., Debnath M. (2018). Role of IL-6/RORC/IL-22 axis in driving Th17 pathway mediated immunopathogenesis of schizophrenia. Cytokine.

[106] Tian T., Wu J., Chen T., Li J., Yan S., Zhou Y., Peng X., Li Y., Zheng N., Cai A. (2022). Long-term follow-up of dynamic brain changes in patients recovered from COVID-19 without neurological manifestations. J. Clin. Investig..

[107] Dong Y., Hu C., Huang C., Gao J., Niu W., Wang D., Wang Y., Niu C. (2021). Interleukin-22 Plays a Protective Role by Regulating the JAK2-STAT3 Pathway to Improve Inflammation, Oxidative Stress, and Neuronal Apoptosis following Cerebral Ischemia-Reperfusion Injury. Mediat. Inflamm..

[108] Sfera A., Andronescu L., Britt W.G., Himsl K., Klein C., Rahman L., Kozlakidis Z. (2023). Receptor-Independent Therapies for Forensic Detainees with Schizophrenia–Dementia Comorbidity. Int. J. Mol. Sci..

[109] Yang T., Wan R., Tu W., Avvaru S.N., Gao P. (2024). Aryl hydrocarbon receptor: Linking environment to aging process in elderly patients with asthma. Chin. Med. J..

[110] Coronas V., Arnault P., Jégou J.-F., Cousin L., Rabeony H., Clarhaut S., Harnois T., Lecron J.-C., Morel F. (2023). IL-22 Promotes Neural Stem Cell Self-Renewal in the Adult Brain. Stem Cells.

[111] Zaharie R.D., Popa C., Schlanger D., Vălean D., Zaharie F. (2022). The Role of IL-22 in Wound Healing. Potential Implications in Clinical Practice. Int. J. Mol. Sci..

[112] Markota A., Endres S., Kobold S. (2018). Targeting interleukin-22 for cancer therapy. Hum. Vaccines Immunother..

[113] Ponce D.M., Alousi A.M., Nakamura R., Slingerland J., Calafiore M., Sandhu K.S., Barker J.N., Devlin S., Shia J., Giralt S. (2023). A phase 2 study of interleukin-22 and systemic corticosteroids as initial treatment for acute GVHD of the lower GI tract. Blood.

[114] Serafim B., Bernardino A.R., Freitas F., Torres C.A.V. (2023). Recent Developments in the Biological Activities, Bioproduction, and Applications of *Pseudomonas* spp. Phenazines. Molecules.

[115] Modoux M., Rolhion N., Lefevre J.H., Oeuvray C., Nádvorník P., Illes P., Emond P., Parc Y., Mani S., Dvorak Z. (2022). Butyrate acts through HDAC inhibition to enhance aryl hydrocarbon receptor activation by gut microbiota-derived ligands. Gut Microbes.

[116] Cha J.W., Lee S.I., Kim M.C., Thida M., Lee J.W., Park J.-S., Kwon H.C. (2015). Pontemazines A and B, phenazine derivatives containing a methylamine linkage from Streptomyces sp. UT1123 and their protective effect to HT-22 neuronal cells. Bioorg. Med. Chem. Lett..

[117] Ooi S.L., Green R., Pak S.C. (2018). N-Acetylcysteine for the Treatment of Psychiatric Disorders: A Review of Current Evidence. Biomed Res Int..

[118] Kim M.-H., Kim H. (2017). The Roles of Glutamine in the Intestine and Its Implication in Intestinal Diseases. Int. J. Mol. Sci..

[119] Kim H., Jeong S., Kim S.W., Kim H.J., Kim D.Y., Yook T.H., Yang G. (2024). Indigo Naturalis in Inflammatory Bowel Disease: Mechanisms of action and insights from clinical trials. J. Pharmacopunct..

[120] Linck V.M., Herrmann A.P., Piato A.L., Detanico B.C., Figueiró M., Flório J., Iwu M.M., Okunji C.O., Leal M.B., Elisabetsky E. (2011). Alstonine as an antipsychotic: Effects on brain amines and metabolic changes. Evid. Based Complement. Altern. Med..

[121] Schwartz D.L. (2016). Quercetin as an Augmentation Agent in Schizophrenia. J. Clin. Psychopharmacol..

[122] Dinakaran D., Sreeraj V.S., Venkatasubramanian G. (2022). Role of Curcumin in the Management of Schizophrenia: A Narrative Review. Indian J. Psychol. Med..

[123] Ji X., Chai J., Zhao S., Zhao Y. (2025). Plant-derived polyphenolic compounds for managing schizophrenia: Mechanisms and therapeutic potential. Front. Pharmacol..

[124] Zhou H., Liang H., Li Z.F., Xiang H., Liu W., Li J.G. (2013). Vagus nerve stimulation attenuates intestinal epithelial tight junctions disrup-tion in endotoxemic mice through α7 nicotinic acetylcholine receptors. Shock.

[125] Lv S., Luo C. (2024). Ferroptosis in schizophrenia: Mechanisms and therapeutic potentials (Review). Mol. Med. Rep..

[126] Xu M., Tao J., Yang Y., Tan S., Liu H., Jiang J., Zheng F., Wu B. (2020). Ferroptosis involves in intestinal epithelial cell death in ulcerative colitis. Cell Death Dis..

[127] Peng X., Yan H., You Z., Wang P., Wang S. (2004). Effects of enteral supplementation with glutamine granules on intestinal mucosal barrier function in severe burned patients. Burns.

[128] Ding L.-A., Li J.-S. (2003). Effects of glutamine on intestinal permeability and bacterial translocation in TPN-rats with endotoxemia. World J. Gastroenterol..

[129] Jiao N., Wu Z., Ji Y., Wang B., Dai Z., Wu G. (2015). L-Glutamate Enhances Barrier and Antioxidative Functions in Intestinal Porcine Epithelial Cells. J. Nutr..

[130] Madeira C., Alheira F.V., Calcia M.A., Silva T.C.S., Tannos F.M., Vargas-Lopes C., Fisher M., Goldenstein N., Brasil M.A., Vinogradov S. (2018). Blood Levels of Glutamate and Glutamine in Recent Onset and Chronic Schizophrenia. Front. Psychiatry.

[131] Deters B.J., Saleem M. (2021). The role of glutamine in supporting gut health and neuropsychiatric factors. Food Sci. Hum. Wellness.

[132] Silva-Parra J., Ramírez-Martínez L., Palafox-Gómez C., Sandu C., López-Bayghen E., Vega L., Elizondo G., Loaeza-Loaeza J., Hernández-Sotelo D., Hernández-Kelly L.C. (2024). Aryl Hydrocarbon Receptor Involvement in the Sodium-Dependent Glutamate/Aspartate Transporter Regulation in Cerebellar Bergmann Glia Cells. ACS Chem. Neurosci..

[133] Naganuma M. (2019). Treatment with indigo naturalis for inflammatory bowel disease and other immune diseases. Immunol. Med..

[134] Kumar H., Datusalia A.K., Kumar A., Khatik G.L. (2025). Network pharmacology exploring the mechanistic role of indirubin phytoconstituent from *Indigo naturalis* targeting GSK-3 *β* in Alzheimer’s disease. J. Biomol. Struct. Dyn..

[135] Weathington N.M., Snavely C.A., Chen B.B., Zhao J., Zhao Y., Mallampalli R.K. (2014). Glycogen Synthase Kinase-3β Stabilizes the Interleukin (IL)-22 Receptor from Proteasomal Degradation in Murine Lung Epithelia. J. Biol. Chem..

[136] Yasmin S., Yousaf M., Majeed K., Rashid M., Tahir S., Numan M., Mustafa R., Nagra S., Zaneb H., Rehman H. (2021). Dietary Catharanthus roseus modulates intestinal microarchitecture in broilers. S. Afr. J. Anim. Sci..

[137] Linck V.M., Ganzella M., Herrmann A.P., Okunji C.O., Souza D.O., Antonelli M.C., Elisabetsky E. (2015). Original mechanisms of antipsychotic action by the indole alkaloid alstonine (*Picralima nitida*). Phytomedicine.

[138] Wang X., Xie X., Li Y., Xie X., Huang S., Pan S., Zou Y., Pan Z., Wang Q., Chen J. (2023). Quercetin ameliorates ulcerative colitis by activating aryl hydrocarbon receptor to improve intestinal barrier integrity. Phytother. Res..

[139] Suzuki T., Hara H. (2009). Quercetin Enhances Intestinal Barrier Function through the Assembly of Zonnula Occludens-2, Occludin, and Claudin-1 and the Expression of Claudin-4 in Caco-2 Cells. J. Nutr..

[140] Amasheh M., Schlichter S., Amasheh S., Mankertz J., Zeitz M., Fromm M., Schulzke J.D. (2008). Quercetin Enhances Epithelial Barrier Function and Increases Claudin-4 Expression in Caco-2 Cells3. J. Nutr..

[141] Page C.E., Coutellier L. (2018). Reducing inhibition: A promising new strategy for the treatment of schizophrenia. eBioMedicine.

[142] Samini M. (2019). The Neuro-Protective effects of Quercetin. Res. J. Pharm. Technol..

[143] Tian J., Kaufman D.L. (2023). The GABA and GABA-Receptor System in Inflammation, Anti-Tumor Immune Responses, and COVID-19. Biomedicines.

[144] Mehany A.B.M., Belal A., Santali E.Y., Shaaban S., Abourehab M.A.S., El-Feky O.A., Diab M., Galala F.M.A.A., Elkaeed E.B., Abdelhamid G. (2022). Biological Effect of Quercetin in Repairing Brain Damage and Cerebral Changes in Rats: Molecular Docking and In Vivo Studies. BioMed Res. Int..

[145] Fan H.-R., Du W.-F., Zhu T., Wu Y.-J., Liu Y.-M., Wang Q., Wang Q., Gu X., Shan X., Deng S. (2018). Quercetin Reduces Cortical GABAergic Transmission and Alleviates MK-801-Induced Hyperactivity. eBioMedicine.

[146] Otimenyin S.O., Ior L.D. (2021). Medicinal Plants Used in the Management of Psychosis. Complementary Therapies.

[147] Enayati A., Soghi A., Butler A.E., Rizzo M., Sahebkar A. (2023). The Effect of Curcumin on the Gut-Brain Axis: Therapeutic Implications. J. Neurogastroenterol. Motil..

[148] Miodownik C., Lerner V., Kudkaeva N., Lerner P.P., Pashinian A., Bersudsky Y., Eliyahu R., Kreinin A., Bergman J. (2019). Curcumin as Add-On to Antipsychotic Treatment in Patients with Chronic Schizophrenia: A Randomized, Double-Blind, Placebo-Controlled Study. Clin. Neuropharmacol..

[149] Hosseininasab M., Zarghami M., Mazhari S., Salehifar E., Moosazadeh M., Fariborzifar A., Babaeirad S., Hendouei N. (2021). Nanocurcumin as an Add-on to Antipsychotic Drugs for Treatment of Negative Symptoms in Patients with Chronic Schizophrenia. J. Clin. Psychopharmacol..

[150] Feng J., Zheng T., Hou Z., Lv C., Xue A., Han T., Han B., Sun X., Wei Y. (2020). Luteolin, an aryl hydrocarbon receptor ligand, suppresses tumor metastasis in vitro and in vivo. Oncol. Rep..

[151] Sahn B., Pascuma K., Kohn N., Tracey K.J., Markowitz J.F. (2023). Transcutaneous auricular vagus nerve stimulation attenuates inflammatory bowel disease in children: A proof-of-concept clinical trial. Bioelectron. Med..

[152] Bonaz B. (2022). Anti-inflammatory effects of vagal nerve stimulation with a special attention to intestinal barrier dysfunction. Neurogastroenterol. Motil..

[153] Soylu A., Alim E., Dizakar S.O.A., Atalar K., Bahcelioglu M. (2025). Is Transauricular Vagus Nerve Stimulation the Spark for Modulation of Synaptic Alterations in Ulcerative Colitis?. Bratisl. Med. J..

[154] Corsi-Zuelli F.M.d.G., Brognara F., da Silva Quirino G.F., Hiroki C.H., Fais R.S., Del-Ben C.M., Ulloa L., Salgado H.C., Kanashiro A., Loureiro C.M. (2017). Neuroimmune Interactions in Schizophrenia: Focus on Vagus Nerve Stimulation and Activation of the Alpha-7 Nicotinic Acetylcholine Receptor. Front. Immunol..

[155] Gargus M., Ben-Azu B., Landwehr A., Dunn J., Errico J.P., Tremblay M. (2025). Mechanisms of vagus nerve stimulation for the treatment of neurodevelopmental disorders: A focus on microglia and neuroinflammation. Front. Neurosci..

[156] Liu Y., Pang Y., Liu Y., Zhang N., Mi L., Gao Y., Gao W., Xu K. (2025). Low-dose rituximab in lupus enteritis: A comparative study on its efficacy in modulating mucosal immunity and reducing inflammation. Arthritis Res. Ther..

[157] Humble M.B., Eklund D., Fresnais D., Hylén U., Sigra S., Thunberg P., Bejerot S. (2023). Rituximab for treatment-resistant schizophrenia and/or obsessive-compulsive disorder (OCD): Functional connectivity and cytokines associated with symptomatic improvements. Eur. Psychiatry.

[158] Buchanan R.W., Werkheiser A.E.M., Michel H.B., Zaranski J.M., Glassman M., Adams H.A.P., Vyas G.D., Blatt F., Pilli N.R., Pan Y. (2024). Prebiotic Treatment in People with Schizophrenia. J. Clin. Psychopharmacol..

[159] Marinelli L., Martin-Gallausiaux C., Bourhis J.-M., Béguet-Crespel F., Blottière H.M., Lapaque N. (2019). Identification of the novel role of butyrate as AhR ligand in human intestinal epithelial cells. Sci. Rep..

[160] Bakshi J., Mishra K. (2024). Sodium butyrate prevents lipopolysaccharide induced inflammation and restores the expression of tight junction protein in human epithelial Caco-2 cells. Cell. Immunol..

[161] Lühder F., Kebir H., Odoardi F., Litke T., Sonneck M., Alvarez J.I., Winchenbach J., Eckert N., Hayardeny L., Sorani E. (2017). Laquinimod enhances central nervous system barrier functions. Neurobiol. Dis..

[162] Sun J., Shen X., Dong J., Zhao J., Zuo L., Wang H., Li Y., Zhu W., Gong J., Li J. (2015). Laquinimod ameliorates spontaneous colitis in interleukin-10-gene-deficient mice with improved barrier function. Int. Immunopharmacol..

[163] Mishra M.K., Wang J., Keough M.B., Fan Y., Silva C., Sloka S., Hayardeny L., Brück W., Yong V.W. (2014). Laquinimod reduces neuroaxonal injury through inhibiting microglial activation. Ann. Clin. Transl. Neurol..

